# A WeChat-based competency and performance checklist in basic surgical skills course for military medical academy undergraduates

**DOI:** 10.1186/s12909-022-03939-x

**Published:** 2022-12-12

**Authors:** Pengfei Luo, Xianqi Shui, Yamei Zhou, Xiaoyu Jiang, Jia Liu, Yu Sun, Yifan Chang

**Affiliations:** 1 Department of Burn Surgery, Changhai Hospital, Naval Medical University, Shanghai, 200433 China; 2 Department of Medical Education – Surgery and Battlefield Surgery, Changhai Hospital, Naval Medical University, Shanghai, 200433 China; 3 Department of Urology, Changhai Hospital, Naval Medical University, Shanghai, 200433 China

**Keywords:** Checklist, Basic surgical skills, Undergraduate, WeChat, Competency, Performance, Evaluation of teaching

## Abstract

**Background:**

Basic surgical skills (BSS) is a key bridging course for medical students to acquire basic surgical maneuvers and practice animal surgery before clinical rotation, but the complexity of operational procedures and high demands on asepsis may lead to poor performance and frequent error during practice. The current study intended to improve BSS teaching outcomes by implementing smartphone app-based competency and performance checklists for medical academy undergraduates.

**Methods:**

WeChat-based checklists containing competency and performance modules were designed, distributed and collected via smartphone. One hundred seventy-six third-grade undergraduate cadets majoring in clinical medicine or anesthesiology were prospectively enrolled, with 92 set as study group and 84 as control group. Checklists were distributed for self-evaluation before and after each class throughout the semester of autumn 2021-2022. Student age, previous Grade Point Average (GPA), average grades of BSS (including grade-A rate and pass rate), operative time, error rate, and perioperative complications of intestinal anastomosis performed on Beagle dogs between the groups were compared to evaluate the efficacy of the checklists.

**Results:**

The students aged 20.2 ± 0.63 in Group A and 20.3 ± 0.92 in Group B (*P* = 0.15), with a previous GPA of 2.9 ± 0.61 vs. 2.87 ± 0.58 (*P* = 0.61). The average operative time on their final lesson of intestinal anastomosis was 192.3 ± 27.18 min vs. 213.8 ± 29.48 min (*P* < 0.001). All students passed in BSS course, with a final grade of 89.45 ± 4.360 in Group A and 86.64 ± 4.026 in Group B (*P* < 0.001), in which grade-A rate was 46.7% vs. 26.2% (*P* = 0.005). For perioperative comorbidities, 4/23 (17.4%) animals in Group A and 5/21 (23.8%) in Group B recorded wound dehiscence or other incision-related complications; no animals died in Group A, and 2 died in Group B due to hemorrhagic shock or sepsis.

**Conclusions:**

The implementation of WeChat-based checklist is a reflection of improved quality of teaching in BSS course that may promote the students’ competency and performance.

**Supplementary Information:**

The online version contains supplementary material available at 10.1186/s12909-022-03939-x.

## Background

Basic surgical skills (BSS), comprising of basic knot-tying and suturing practiced on suturing models, and performing general surgeries like splenectomy or typhlectomy on experimental animals, is an indispensable bridging course widely adopted in medical school curricula or residency programs worldwide [1, 2]. In China, BSS is generally designed for third to fourth-grade undergraduate medical students majoring in 5-year or 8-year clinical medicine, to translate their theoretical knowledge to practical hands-on skills before they are allowed to operate on a patient during clinical rotation. BSS focuses both on technical competency and on the actual surgical planning, intraoperative problem-solving, team coordination, as well as following principles of asepsis and providing patient care on a live animal model. Because essentially a large portion of the course grade depends on students’ performance to actually carry out a basic animal surgery by themselves as a surgical team, it is crucial for the students themselves, rather than the faculty, to obtain a periodical self-evaluation and be fully aware of their competency level during the course; also various new teaching methods such as telestration [3], virtual classroom [4], or peer-teaching [5], have been proposed in BSS courses by a number of studies.

The current study intended to improve BSS teaching outcomes by implementing a WeChat-based competency and performance checklists for medical academy undergraduates. Its efficacy and value for evaluation of teaching have been investigated.

## Methods

### Design of checklist

**Fig. 1:**
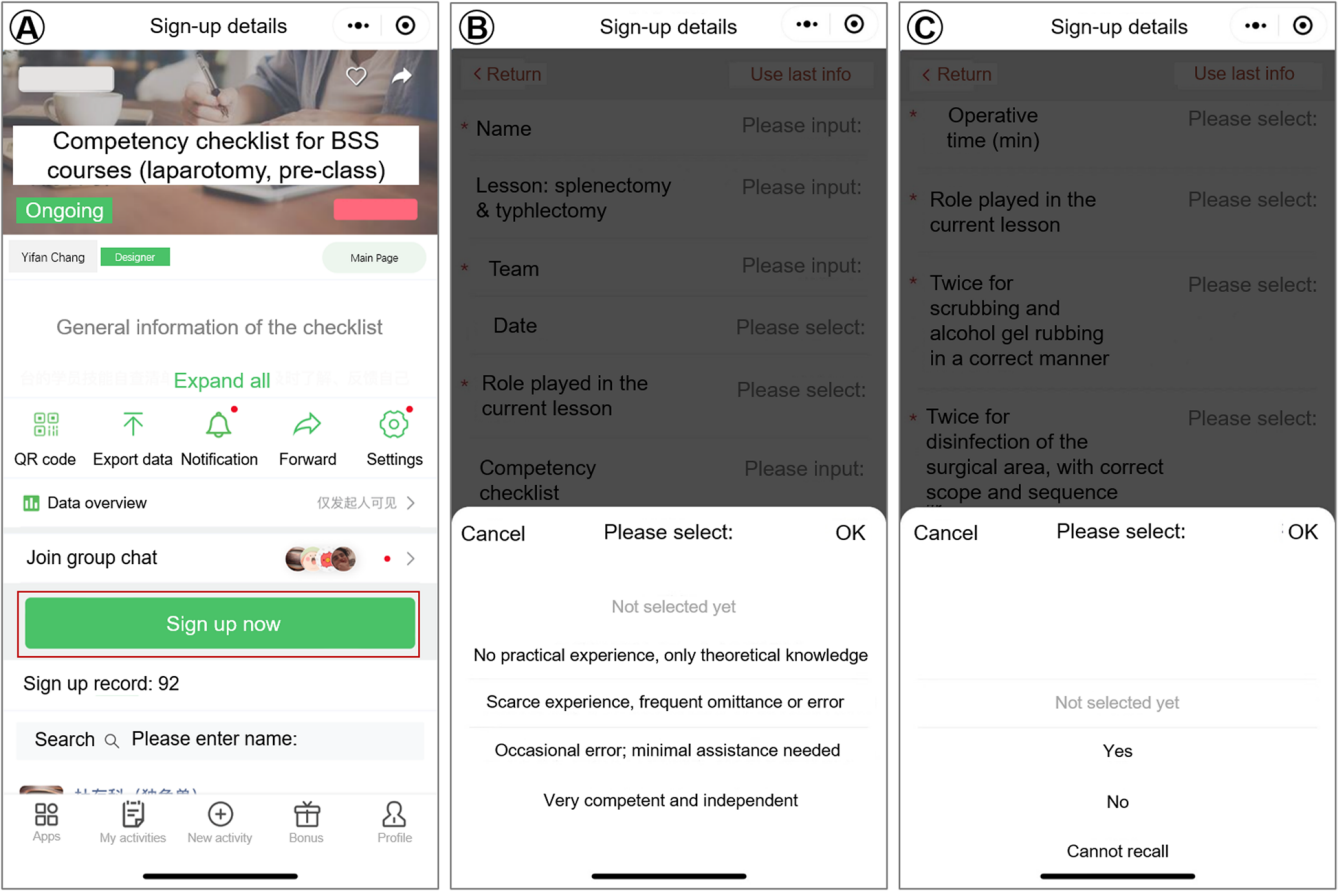
Distribution, collection, and interface of the checklist on WeChat. **A** an overall profile of the checklist with general information and “click to sign up now” button for the respondents, as well as function of generating QR code, export to Microsoft Excel, sending notifications, forward and sharing, and sign up record for the investigators; **B** template of a competency checklist for respondents to select the relevant information; **C** template of a performance checklist

The checklist we designed for the present study was a smart phone-based online form for respondents to fill out basic information and check specific questions concerning specific surgical skills or procedures. It contained two modules, the competency checklist, which was for pre-class self-awareness of how proficient they can carry out a specific surgical skill (e.g., different suturing methods), and the performance checklist, which was for after-class evaluation of how good they have actually performed in the surgical procedures, in regard of surgical time, error occurred throughout the operation, and perioperative comorbidities (Table 1). Each module contained 10-15 task-specific items according to the different content of different lessons. The checklists were distributed and collected with a sign-up mini-program on WeChat (Version 8.0, Tencent Technology, Shenzhen, China) (Fig. 1).

**Table 1:** An example of basic surgical skills checklist, comprising of basic information, competency checklist for pre-class self-evaluation, and performance checklist for after-class team debriefing. The checklist is designed and imported to WeChat for further distribution and collection of data

Basic surgical skills competency and performance checklist					
Name		Team#		Date	
Lesson	**Splenectomy & typhlectomy**	Role in current lesson			
1.Competency checklist (Pre-class self-evaluation)					
1 = No practical experience, only theoretical knowledge 2 = Scarce experience, frequent omittance or error 3 = Occasional error; minimal assistance needed 4 = Very competent and independent					
	**Basic surgical skills**	**1**	**2**	**3**	**4**
1	Correct Identification and use of surgical instruments				
2	Bare-handed knot-tying				
3	Knot-tying with instruments				
4	Simple interrupted suture				
5	Continuous suture				
6	Interrupted vertical mattress				
7	Interrupted horizontal mattress				
8	Continuous horizontal mattress				
9	Purse-string suture				
10	Tissue ligation				
11	Scrubbing				
12	Asepsis and draping				
2. Performance checklist (After-class self-evaluation)					
Please evaluate the performance of **your TEAM** and check the boxes below:					
	**Performance**		Yes	No	Cannot recall
1	Correct scrubbing and alcohol rubbing				
2	Correct disinfection and draping by the 1st assistant				
3	Correct gown dressing and glove wearing				
4	Correct setup of the instrument table by the scrub nurse				
5	Correct use and handling of surgical instruments and sutures				
6	Proper dissection and ligation of splenic vessels and ligaments				
7	Proper stump burying of the cecum with purse string				
8	Correct count of sponges, needles and instruments				
9	All procedures completed				
10	No violation of asepsis				
11	No major complications occurred				
12	No sharps injury occurred				
13	Experimental animal remained vitally stable				
14	Adequate surgical planning and teamwork

### Distribution, collection and evaluation of data

In this prospective study, BSS was offered to 176 third-grade undergraduate students of ‘19 from Naval Medical University, Shanghai, China, in the autumn semester of 2021-22, in which 92 (majoring in 5-year clinical medicine) were in Class A and set as study group, and 84 (62 majoring in 5-year clinical medicine + 22 majoring in 5-year anesthesiology) were in Class B and set as control group (Additional file 1). Both classes had identical study materials, course duration, instructor guidance, and examination standard. The final grade was appraised upon their performance on the final lesson to carry out intestinal resection and anastomosis on animal models in regard of skills proficiency, error, teamwork, and surgical outcomes. Distribution of the checklist was blinded to Class B. Faculty members involved in teaching, tutoring, performance appraisal, and grading, were also blinded from the existence of the checklists. Student age, previous Grade Point Average (GPA, 4-point scale), operative time, occurrence of error and surgery-related comorbidities (based on Clavien-Dindo classification system), and final grade (100-point scale, including Grade-A rate (≥ 90 points) and pass rate (≥ 60 points)), were collected for data analysis. Student’s critical review on the checklist and their satisfaction feedbacks were also collected.

### Procurement, anesthesia and euthanasia of experimental animals

Animal surgery was carried out on 44 beagle dogs (41 male and 3 female), purchased from Jiagan Biotech Co., Ltd. (Shanghai, China), in which 23 were randomly distributed to study group and 21 to control group, according to the number of students in the two groups, who were grouped into 4 for each animal. The dogs aged 4.0 ± 1.39 months and weighed 6.2 ± 1.60 kg (mean ± SD), that were immuned with Rabies Virus, Canine Parvovirus, Canine Distemper Virus, Infectious Cercopithecine Herpesvirus Type 1 before delivery. Intramuscular injection of xylazine hydrochloride (2 mg/kg, Huamu Animal Healthcare Product Co, Ltd. (Changchun, Jilin, China)) was adopted for general anesthesia. After each course, the dogs were sent back to the animal center of our university to continue housing and husbandry, in which individual caging, daily hygiene, sunlight, feeding, noise reduction, and daily health monitoring was offered. The experimental animals were reused for the entire semester. In case of postoperative comorbidities, respective treatment was offered accordingly; should severe intraoperative complications leading to inevitable death be encountered, euthanasia was delivered with intravenous pentobarbital sodium (150 mg/kg, Civi Chemical Technology Co., Ltd. (Shanghai, China)), followed by cremation of the body. By the end of the study, animals that were still healthy were sent to local animal welfare agencies for adoption.

### Data analysis

Data were exported to Microsoft Excel 2016 (Microsoft Corporation, Redmond, USA) and analyzed with STATA 15 (StataCorp, College Station, TX, USA). T-test was used to compare numerical variables (i.e., age, GPA, operative time, and final grade) between the two groups. Pearson’s chi-square test was used to compare categorical variables (Grade-A rate, pass rate, and perioperative complications).

## Results

Baseline and perioperative outcomes were listed in Table 2. The students aged 20.2 ± 0.63 in Group A and 20.3 ± 0.92 in Group B (mean ± SD, *P* = 0.15), with a previous GPA of 2.9 ± 0.61 vs 2.87 ± 0.58 (mean ± SD, *P* = 0.61). Overall response rate of the checklist was 96.6%. After distribution of checklists throughout the semester, the average operative time on their final lesson of intestinal anastomosis was 192.3 ± 27.18 min vs 213.8 ± 29.48 min (mean ± SD, *P* < 0.001). The final grade was 89.45 ± 4.360 out of 100 in Group A and 86.64 ± 4.026 out of 100 in Group B (*P* < 0.001), in which grade-A rate was 46.7% vs 26.2% (*P* = 0.005). All students passed in this course. For perioperative comorbidities, 4/23 (17.4%) animals in Group A and 5/21 (23.8%) in Group B recorded wound dehiscence, infection, or other forms of incision-related complications; no animals died in Group A till postoperative day 7, and 2 died in Group B, in which 1 died on table due to unidentified intra-abdominal bleeding that led to hemorrhagic shock, and 1 died on postoperative day 4 due to sepsis; however, no statistical significance was reached (*P* = 0.20 and *P* = 0.13, respectively). For feedback of the checklist, 70.8% believed that the checklist was highly helpful and 24.7% reported somewhat helpful in terms of acquiring skills and improving proficiency, and 94.4% believed that checklists stimulated their enthusiasm for learning. 83.1% recommended the use of checklist in BSS or other clinical courses.

**Table 2:** Baseline and perioperative outcomes

	Study group	Control	***P***
**N**	92	84	
**Age (yrs, mean ± SD)**	20.2 ± 0.63	20.3 ± 0.92	0.15
**Previous GPA (mean ± SD)**	2.90 ± 0.61	2.87 ± 0.58	0.61
**Operative time (min, mean ± SD)**	192.3 ± 27.18	213.8 ± 29.48	< 0.001
**Final Grade (mean ± SD)**	89.45 ± 4.36	86.64 ± 4.03	< 0.001
**Grade-A rate (%)**	46.7	26.2	0.005
**Pass rate (%)**	100	100	
**Complications (n, %)**			
**< Grade III**	4 (17.4)	5 (23.8)	0.20
**≥ Grade III**	0 (0.0)	2 (9.5)	0.13

## Discussion

Checklists were initially designed for pilots for aircraft configuration more than 80 years ago, in order to acquire in-time remarks for complex and forgetful steps throughout aviation activities. Implementation of the aircraft checklist significantly reduced human error and accident by nearly 80% [6], and was instantly popularized in various industries. In medical practice, perhaps the most successful implementation is the surgical safety checklist proposed by A. Gawande [7] in 2009. Tested in 8 hospitals of different scales in both developed and developing countries, surgical safety checklist showed consistent efficacy in reducing mortality and complication, and has now been recognized by the world health organization (WHO) as a standard in operation room safety management guidelines.

Checklists have shown potential value and benefit for human intensive and multi-step procedures with high complexity, so as to promote consistency and completeness to carry out specific tasks. Apart from surgical procedures, it has also been adopted in nursing skills training [8, 9], emergency life support training [10, 11], and in other forms of medical teaching activities [12]. These studies share one concept in common, that is, although the content of the checklist is designed by investigators or instructors, the checklist yield benefit and values for users themselves by means of self-evaluation and self-reflection, which is in accordance with the intention of our study. Because the students attending the BSS course are Grade 3-4 undergraduates, the fact that they typically lack a solid foundation of clinical knowledge with very limited concept of asepsis, which may lead to frequent error and poor performance, prompted us to design these checklists.

The checklist comprises two modules, the pre-class competency checklist, and the after-class performance checklist. The former is intended to offer the students a general concept of how well they master different basic skills that are necessary for a certain surgical procedure, which helped gain awareness on intensifying pre-class training on skills less competent. The after-class performance checklist, on the other hand, serves as a timely debriefing of procedural correctness, completeness, errors, and team coordination outcomes, which provided feedback for each team members what to pay attention to on their next assignments. In the present study, baseline data such as age and previous GPA between the groups showed no significant difference, showing that the students in the two groups had similar learning competence. After implementation of the checklists over a semester, the benefit we observed was incremental, meaning that the more extensive use of the checklists, the better they will perform. By the end of the semester, the majority of students in Group A felt more confident with markedly improved proficiency, and they believed that the general surgical planning and teamwork were also improved. This is also reflected by a higher average grade (89.45 ± 4.36 vs. 86.64 ± 4.03, *P* < 0.001), grade-A rate (46.7% vs. 26.2%, *P* = 0.005), and significantly shorter operative time (192.3 ± 27.18 vs. 213.8 ± 29.48, *P* < 0.001) on their final examinations. No death occurred in Group A with fewer incision-related complications observed; although no statistical significance was reached, we still believed that it served as a reflection that further supported the potential value of the checklist.

The intention of the checklist was not to serve as a grading tool for teachers by analyzing the percentage of different choices made in each items, but more of a motivation and reminder for conceptual intensification and everyday training for students. The fact that 70.8% regarded the checklist as highly helpful in skills acquisition and proficiency, 94.4% believed that the checklists boosted their motivation to learn, and 83.1% recommended the use of checklist in BSS or other courses, is also a strong support of this idea. Additionally, the use of WeChat as data distributor is another highlight of the study. Being a must-have app with various built-in mini-programs that is compatible with nearly all smart phone operating systems and devices, WeChat is exceptionally high-efficient for both investigators and respondents for data input, distribution, collection and analysis, with minimal cost. Inputting data takes less than 3 mins, and privacy is assured among respondents. These benefits may help increase the respondents’ willingness to participate and provide authentic information per se.

## Conclusions

The implementation of WeChat-based checklist is a reflection of improved quality of teaching in BSS courses, and may promote the students’ competency and performance throughout the course.

## Supplementary Information

**Additional file 1.** Dataset.

## Data Availability

The datasets during and/or analyzed during the current study are attached as Supplementary files.

## Abbreviations

BSS: Basic surgical skills
GPA: Grade point average
SD: Standard deviation
WHO: World Health Organization
